# Use of Spinal Cord Stimulation in Elderly Patients with Multi-Factorial Chronic Lumbar and Non-Radicular Lower Extremity Pain

**DOI:** 10.7759/cureus.1855

**Published:** 2017-11-17

**Authors:** Michelle Granville, Aldo F Berti, Robert E Jacobson

**Affiliations:** 1 Miami Neurosurgical Center, University of Miami Hospital

**Keywords:** spinal cord stimulation, chronic back pain, post laminectomy pain, post joint replacement pain, osteoporotic vertebral compression fractures, peripheral neuropathy pain, failed back surgery syndrome, multiple spinal cord leads, lower extremity pain

## Abstract

Spinal cord stimulation (SCS) is an effective treatment for chronic back and limb pain. The criteria for use of SCS for specific problems such as failed back surgery syndrome (FBSS), peripheral neuropathic pain and residual pain after joint replacement is well established. With an aging population, there are more patients presenting with a combination of various multi-factorial chronic pain problems rather than from a single clear cause. It is not uncommon to see patients with chronic back pain years after spine surgery with new additional pain in the area of joint replacement or due to peripheral neuropathy. In most of these patients, one area is the primary cause of their pain, while the other more secondary. Multiple chronic problems complicate the pain management of the primary cause and also can diminish the effect of SCS that only targets the primary problem. The primary and secondary causes of pain were ranked by the patient including the duration of their chronic pain for each area. This helped establish criteria for use of SCS in these complex pain patients. The patients were evaluated initially with an epidural stimulator trial and if they obtained 50% or greater pain relief to the primary pain generating area, permanent implantation of one or more arrays of spinal cord electrodes was performed but planned to cover also the secondary pain areas. Post-implant follow-up evaluation at one, three and six months included measurement of visual analog scale (VAS), use of pain medication and degree of functional activity and behavior. This report looks at the effectiveness of using multiple overlapping electrodes for SCS in patients with multi-factorial chronic pain.

## Introduction

**Figure 1:**
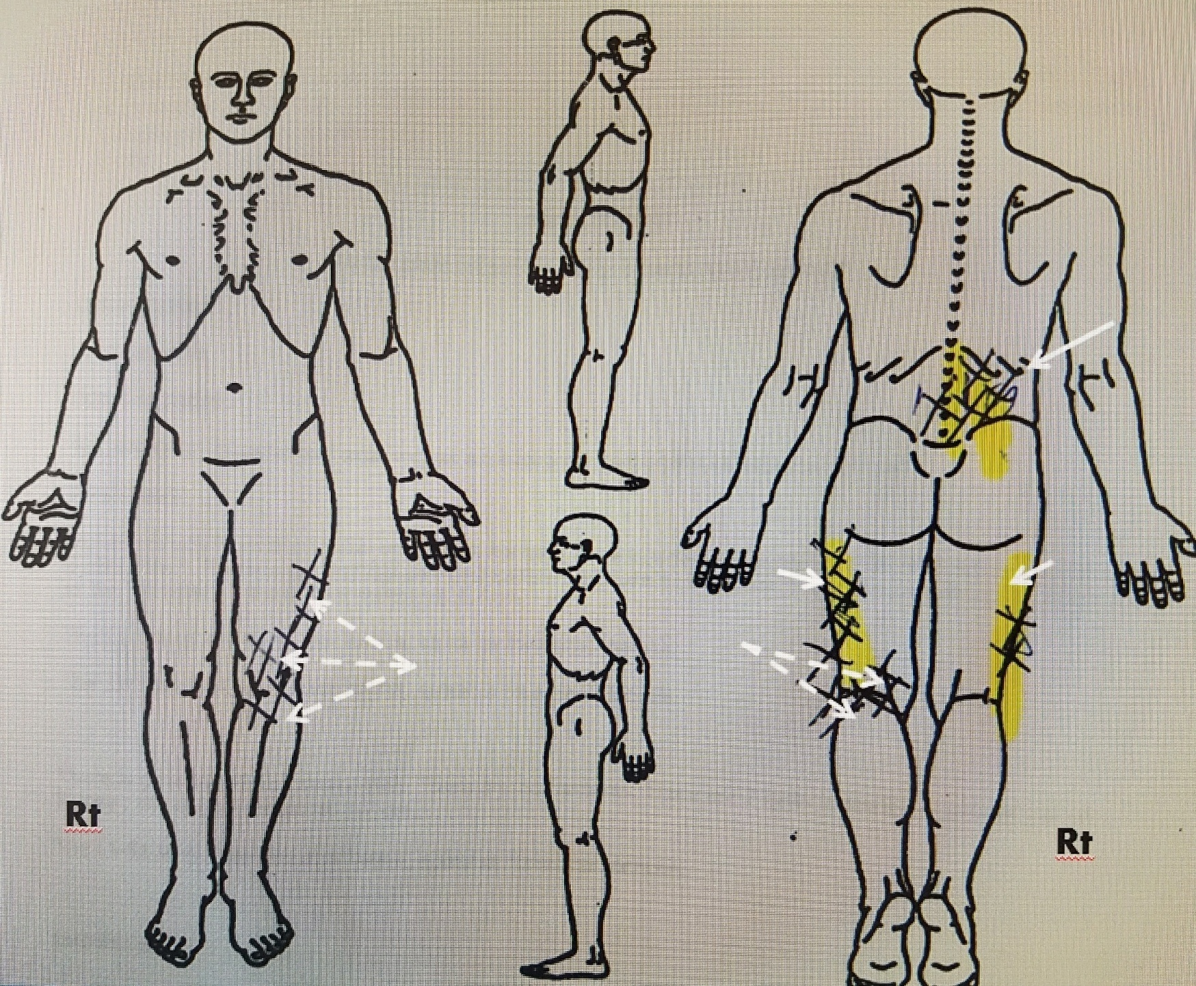
Pain drawing in 73-year-old male with lumbar spondylosis, previous L4-5 laminectomy with pain and two left knee replacements. Anterior and posterior (AP) views with the right side marked (Rt) showing cross hatched areas of pain. The areas in yellow demarcate pain secondary to previous laminectomy. The back pain is more to the right, associated with bilateral lateral posterior thigh pain ( solid white arrows). The patient also had distinct left knee pain after two knee replacement surgeries. The knee pain was both in the posterior knee area, the anterior left knee as well as in the left lateral area above the knee joint (dashed white arrows). He rated pain as 9/10 in both areas. He stated the back pain was constant with mild lateral thigh pain later in the day. The left knee pain was dull but significantly worse with weight bearing, standing and walking.

In reviewing a series of patients being evaluated for a trial of spinal cord stimulation for pain control of chronic back pain after lumbar surgery and also a group of patients with multiple lumbar vertebral fractures, a number of patients were identified that also had non-radiating limb pain that was distinct from residual sciatic radicular pains. These patients, besides axial lumbar pain, had concurrent problems secondary to painful diabetic or vascular peripheral neuropathy or chronic pain from hip or knee replacement. Through both pain diagrams and verbal description, the patients were able to distinguish the differences in the type of pain and overlapping regions of pain (Figure 1).

Studies have shown that 10% to 25% of multilevel spine surgery patients, especially after fusion, have chronic pain unresponsive to medication and physical therapy. Persistent pain with some residual radiculopathy is often grouped into a very broad category labeled 'failed back surgery syndrome' (FBSS). Many patients treated with FBSS get at least 50% pain relief with the implantation of a spinal cord stimulator (SCS) that provides stimulation to the low back and into both legs [1,2]. Large studies of diabetics show that 50% develop peripheral neuropathy and up to 21% eventually get painful neuropathy requiring medical management and a small percentage have implantation of spinal cord stimulators for management of chronic neuropathic pain [3,4]. Vascular insufficiency and the related painful neuropathy due to decreased peripheral arterial flow have been found to respond to SCS [5,6]. Follow-up studies after joint replacement show unfavorable outcome and chronic localized pain limiting activity in 7% to 23% of hip replacements and 10% to 14% of knee replacements [7-9]. Spinal cord stimulation or peripheral nerve field stimulation has been used to treat chronic pain after joint replacement [10]. Some degree of persistent pain after vertebral augmentation and kyphoplasties is common especially when the fractures are in the lumbar spine [11,12]. Back pain persisting after the first three months post vertebroplasty is seen in 19% to 23% of cases [13]. Radicular pain is not usually associated with osteoporotic vertebral compression fractures, however, these elderly patients may also have chronic extremity pain from peripheral neuropathy or from other osteoporotic fractures in the hip or after joint replacement that can be helped with spinal cord stimulation targeting both the lumbar spine and lower extremities [11,13].

There are specific criteria developed for using spinal cord stimulation for treatment of chronic pain for each of the above groups and all protocols include an initial trial implant [1,4,6,10,12]. This report will examine patients that have the concurrent existence of back and limb pain from a primary source such as the low back after surgery and a secondary source of pain from peripheral neuropathy or post joint replacement. There often is significant overlap of primary and secondary causes as well as the regions of pain affecting the back and the lower extremities. Recognizing that multiple different pain generators can affect how and where spinal cord stimulation electrodes are implanted is critical to obtaining the best outcome for chronic pain patients.

## Materials and methods

While evaluating patients in a general neurosurgical practice for post-laminectomy and limb pain it was observed that many elderly patients seen after lumbar spine surgery for persistent lumbar pain, had minimal or no radicular pain but had other lower extremity pain either related to previous hip or knee replacement or lower leg sensory complaints and neuropathic pain from peripheral neuropathy. This non-radicular extremity pain was always a secondary complaint compared to the primary complaint of back pain. All patients rated the secondary limb pain as at least 50% intensity of their overall pain.

To examine the frequency of these mixed causes of leg complaints a comprehensive retrospective chart review of all patients evaluated with a temporary trial of epidural stimulation with the Medtronic^R^ percutaneous leads and permanent implant with a battery (Medtronic^R^, Minneapolis, Minnesota, USA) were re-examined. The underlying conditions causing pain were tabulated by location and ranked by severity. The groups identified included FBSS, peripheral neuropathic pain (PNP), vascular neuropathic pain (VNP), joint replacement pain (JRP) and vertebral compression fracture pain (VCFP). If available, pain drawings and visual analog scale (VAS) scores before and after trial and permanent implantation were used. The type, position and number of epidural stimulation electrodes were compared. Medication use and activity level before and after implant were measured.

## Results

In total, 21 patients were evaluated with spinal cord stimulation with temporary trials and 17 underwent subsequent permanent implantation of a spinal cord stimulator with one or two leads. The implant to trial ratio was 80%. Of the 17 cases receiving permanent implants, the average age was 71.5 years and females made up 70.5% of the group.

There were 11 patients whose primary complaint was related to back pain due to FBSS and 64% were female. The secondary painful areas in the FBSS group consisted of six patients who also had PNP and three with secondary limb pain after JRP. Two patients, both female, had all three with a combination of FBSS, PNP, and JRP pain. Eight patients had peripheral neuropathy, with one patient being non-diabetic, but having a long history of cigarette smoking and peripheral vascular disease. Five patients presented with postoperative pain following joint replacement of the hip or knee. There were six patients being evaluated with SCS for chronic lumbar pain without laminectomy but pain from multiple lumbar VCF. Two patients had pain from previous lumbar surgery compounded by associated lumbar vertebral compression fractures with pain. In this group only one patient also had chronic residual pain from knee replacement and two also had PNP. Twelve cases had two leads placed for better coverage. Patients who presented with more than one of the above-mentioned pathologies did better with two leads for a broader coverage of stimulation. Average preoperative VAS was 8/10. Average three-month follow-up postoperative VAS was 3/10 (Table 1).

**Table 1:** Tabulation of characteristics and different pain areas of the patients. F: Female; M: Male; FBSS: Failed back surgery syndrome; PNP: Peripheral neuropathic pain; JRP: Joint replacement pain; VCFP: Vertebral compression fracture pain; LEADS: the number of epidural leads implanted.

AGE/SEX	FBSS	PNP	JRP	VCFP	LEADS
78 F	yes	yes	no	no	1
77 F	yes	yes	no	no	2
76 M	yes	no	no	yes	2
76 F	yes	no	no	no	2
72 F	yes	no	no	no	1
72 M	yes	no	no	no	1
71 F	yes	no	no	yes	2
81 M	yes	yes	yes	no	2
78 F	yes	no	yes	no	2
63 F	yes	yes	yes	no	1
73 M	yes	yes	no	no	2
74 M	no	yes	yes	no	2
78 F	no	no	yes	no	1
75 F	no	no	no	yes	2
74 F	no	no	yes	yes	2
71 F	no	yes	no	yes	2
74 F	no	yes	no	yes	2

## Discussion

SCS is a useful tool for chronic pain management for specific conditions including FBSS, diabetic and vascular peripheral neuropathy and chronic post-joint implant pain [1-5]. The criteria and long-term effectiveness of SCS in each of these conditions have been established. Good results are considered to be at least a 50% or greater decrease in pain with less medication use and increased activity [1,2]. In a study of over 300 patients treated with SCS, with an eight-year follow-up, the mean age was 54 and 80% were males with a 67% long-term success rate [14]. In our review the average age was 71.5 years, and females made up a larger percentage of the entire group. In the older population, it is common to see patients with more than one medical/surgical problem such as diabetic or peripheral vascular neuropathy and chronic pain after joint replacement that leads to lower extremity pain. This explains the high incidence of comorbidities contributing to the patient's pain in this review. The subjective area of the pain in the lower extremities from diabetic or vascular peripheral neuropathy can overlap areas of residual leg pain after surgery although it is more typically in the lower feet while FBSS pain is seen in the buttock and upper posterio thighs [1,4,6]. In patients with FBSS that have concurrent problems also causing pain in the lower extremities or a joint, it is important to try and get stimulation coverage for these areas both during the trial and when the permanent leads are implanted.

**Figure 2:**
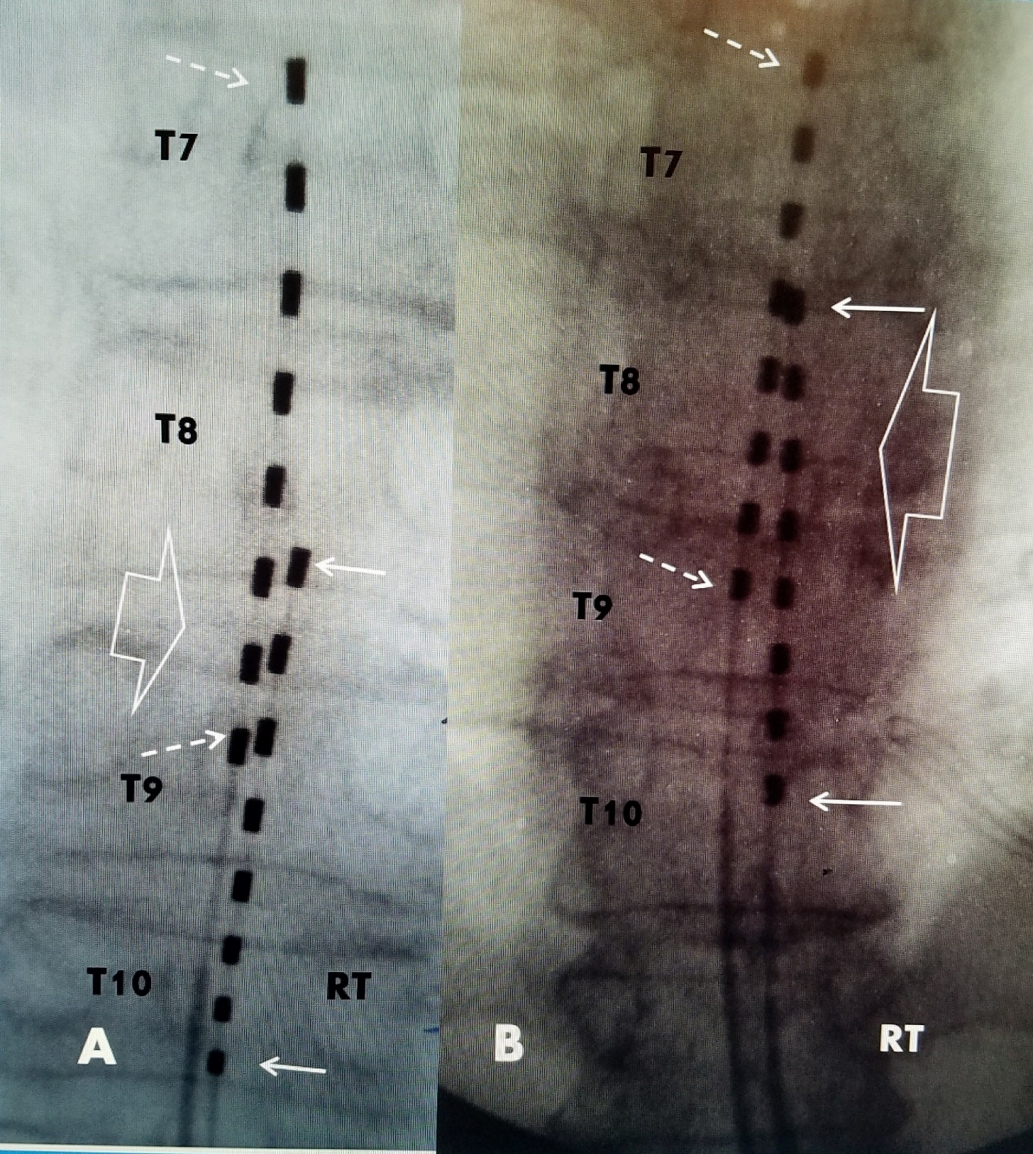
Examples of using overlapping percutaneous leads to get a wider stimulation area. A: Patient with failed back surgery syndrome (FBSS) and painful diabetic peripheral neuropathy affecting both feet. Anterior posterior radiograph showing midline lead extending superiorly from upper T7 to mid T9 (dashed white arrows) and the 2nd midline slightly right lead covers lower T8 thru mid T10 (white arrow). The overlapping three electrodes between T8/9 (open white arrow) allowed bipolar stimulation to the lumbar spine. All leads are centered towards the midline. The second lead from T8/9 to T10/11 provided lower extremity coverage for the peripheral neuropathy. B: Patient with FBSS and right chronic knee pain after joint replacement: Radiograph showing lead positioned in the midline from upper T7 to mid T9 (dashed white arrow) for the lumbar pain. The right lead is parallel from the top T8 to upper T10 (solid white arrow) but more to the right side that provided coverage for the right knee. There is a broad area of five electrodes that overlap from T8 to mid T9 allowing bipolar cross stimulation (open white arrow) for his lumbar pain.

Permanent spinal cord stimulation can be performed with either a paddle lead placed with a thoracic laminotomy or through a percutaneously inserted lead. Studies show slightly more morbidity with paddle leads but better short-term results. However, long-term follow-up at three years shows similar outcomes [15,16]. The results of spinal cord stimulation for FBSS can be affected by the experience and specialty of the implanter [17]. Using a single lead targeting primarily axial lumbar pain may not provide sufficient stimulation coverage for patients who also have peripheral neuropathic pain affecting the lower legs and feet or lateralized pain after joint replacement [14,16,18]. Most battery systems allow the use of two leads which provides the ability to place two separate electrode arrays, one targeting the axial lumbar spine pain and the other targeting the peripheral neuropathic pain or more lateral joint pain. Our experience demonstrates that axial lumbar pain is best covered with electrodes from ZT6-7 to T8-9 while peripheral limb pain requires leads from T8-9 to T10. Localized joint pain is best treated with at least one electrode positioned slightly to the painful side. By overlapping the leads an area of bipolar stimulation can be programmed. Dual or triple array epidural leads have been used to provide stronger axial coverage [19,20]. This may be important in patients with more diffuse lumbar pain associated with multiple vertebral compression fractures [11-13]. Another option is a combination of an epidural lead with a second lead positioned in the lumbar subcutaneous tissue for peripheral nerve field stimulation (PNFS) [21,22]. PNFS can target a localized area, such as the sacroiliac joint, hip or knee joint [9,10,22]. Patients with peripheral neuropathic pain and axial pain from previous lumbar surgery usually need multiple epidural leads [23,24]. Dual epidural leads positioned in a staggered but overlapping manner will provide a specific wider area of stimulation coverage to the axial lumbar spine, allow dual lead cross stimulation and provide additional stimulation coverage to the lower legs for pain secondary to peripheral neuropathy or joint pain (Figure 2).

## Conclusions

Patients presenting with chronic back pain and limb pain after spinal surgery can have lower extremity pain from residual sciatica, but the pain can also be related to peripheral neuropathy or pain after joint replacement. Pain in these areas is distinct from post-laminectomy pain even when the FBSS patient has some residual radicular leg pain. The patient can usually distinguish the different pain areas on close questioning. In an older age group, these additional pain generators causing limb pain must be considered when evaluating a patient either for a spinal cord stimulator trial or placing a permanent stimulator implant. The goal of this review is not to alter the accepted criteria for successful SCS implantation but to highlight that other areas may be contributing to the patient's overall pain. Both during the initial evaluation with a temporary electrode and especially at the time of permanent implantation, the surgeon may consider placing a second array of electrodes that allow programming, currently or on a future date, for broader stimulation coverage of either the affected painful joint or extremities if there is distal pain secondary to peripheral neuropathy.
